# Controlling latent TB tuberculosis infection in high-burden countries: A neglected strategy to end TB

**DOI:** 10.1371/journal.pmed.1002787

**Published:** 2019-04-23

**Authors:** Gavin J. Churchyard, Sue Swindells

**Affiliations:** 1 Aurum Institute, Parktown, South Africa; 2 University of Witwatersrand, School of Public Health, Johannesburg, South Africa; 3 London School of Hygiene and Tropical Medicine, London, United Kingdom; 4 University of Nebraska Medical Center, Omaha, Nebraska, United States of America

## Abstract

In a Perspective, Gavin Churchyard and Sue Swindells discuss the importance of strategies to target latent tuberculosis infection in high risk populations and thus disrupt a reservoir for new infections in high burden countries.

## Latent TB infection: Global burden and risk factors for progressing to TB disease

The World Health Organization (WHO) estimated that 10 million people developed tuberculosis (TB) and 1.6 million died of TB globally in 2017 [1]. In contrast, an estimated 1.7 billion people (23% of the world’s population) are latently infected with TB, from whom cases of active TB disease arise [2]. The greatest burden of TB infection is in WHO Southeast Asia, Western Pacific, and sub-Saharan Africa regions [2]. Controlling the large reservoir of latent TB infection will require finding and treating individuals infected with TB who are otherwise well and are at high risk of progressing to TB disease. Recognising that it will not be possible to end the TB epidemic unless we prevent TB, the United Nations High Level Meeting on TB in September 2018 called on the world to treat a target number of 30 million people living with TB infection. People at highest risk of progressing from latent to active TB disease are those who are immunosuppressed because of HIV or from treatment (e.g., tumour necrosis factor [TNF]-α inhibitors), who are preparing for organ or haematological transplant, who are on dialysis, who are household contacts of patients with pulmonary TB (particularly children <5 years of age), and who have silicosis, which occurs from occupational exposure to silica dust. TB preventive therapy (TPT) entails using one or more antituberculous drugs to treat persons with latent TB infection who are at high risk of progressing to TB disease. In this perspective, we provide the justification for scaling up TPT in high-burden countries.

## TPT

The utility of TPT was demonstrated more than 60 years ago, when isoniazid preventive therapy (IPT) was used to reduce the risk of TB among Alaskan villages, household contacts, and persons living in mental health facilities [3]. Nine to 12 months of IPT substantially reduces the risk of TB among HIV-uninfected adults and children. Among people living with HIV, 6–9 months of IPT substantially reduces the risk of TB regardless of CD4 count or whether they are on antiretroviral therapy or not [4]. IPT is effective among all individuals taking antiretroviral therapy, regardless of whether they have a positive or negative test for TB infection. In high-TB–transmission settings, the protective effect of TPT may wane over time because of ongoing TB transmission. However, the benefit of TPT may be prolonged in these settings by interrupting transmission through case finding and extending the duration of treatment for up to 36 months [5]. Small observational studies suggest that treating presumed multidrug-resistant (MDR) TB infection with appropriate drugs may be effective, but evidence-based data are urgently needed [6]. Three large cluster randomised trials are evaluating the use of levofloxacin, a fluoroquinolone that has been repurposed for treating TB infection (TB CHAMP: ISRCTN92634082, V-QUIN: ACTRN12616000215426), and delamanid, a new TB drug (nitroimidazole) (A5300B/I2003B/PHOENIx: NCT03568383), for treating household contacts exposed to MDR TB patients in high-burden countries.

Scaling up TPT benefits not only individuals but communities as well. In the pre-HIV era, IPT offered to households in Alaska, housing blocks in Tunisia, and villages in Greenland was associated with a decline in TB rates at a population level [3]. More recently, a strategy promoting testing for TB infection and provision of IPT in HIV clinics in Rio de Janeiro reduced the risk of TB or death at the clinic level by 31% [7].

Despite the strong evidence base and the existence of WHO guidelines since 1998 recommending TPT, there has been very limited scale-up of IPT for people living with HIV and child contacts in high-burden countries, apart from South Africa [8,9]. Some of the reasons for the low uptake in high-burden countries include concerns about poor screening tools to exclude active TB disease before starting TPT and the long duration of treatment (6 months up to at least 36 months) [8]. Perceptions among healthcare workers that IPT can cause substantial liver toxicity (based on case reports from the 1970s) and will generate drug-resistant TB if active TB disease is not properly excluded further hampered scale-up of IPT [10,11]. However, studies have shown that these fears are unfounded [11,12].

Appropriate medication formulations may be lacking for children, and uncertainty exists about treatment of pregnant women. Lack of clear guidelines and integration into HIV and child programs, limited access to tests of infection, little demand from people living with HIV and communities, and the risk of reinfection in high-transmission settings have further contributed to the poor scale-up of IPT.

A TB prevention cascade analysis identified major gaps in implementing TPT among adults and children requiring TPT in high- and low-income countries. The biggest gaps in the cascade were completion of testing, completion of medical evaluation, offering treatment, and completion of treatment [13]. Primary healthcare facilities in high-burden countries can readily address the gaps in the TB prevention cascade using a plan, study, do, act approach and using innovative models of delivery, such as community-based TPT provision.

New, shorter regimens herald a new era for TPT. In 2018, WHO issued consolidated guidelines for the programmatic management of latent TB infection, which included new recommendations for the use of short-course, rifamycin-based TPT regimens in high-burden settings [9]. High-dose isoniazid and rifapentine given weekly for 3 months (referred to as 3HP) is recommended for adults and children >2 years of age, and 3 months of daily isoniazid and rifampicin (available as a fixed-dose combination tablet or dispersible tablet) is recommended for children and adolescents <15 years of age [9]. The short-course regimens address some of the barriers to implementing IPT in that they are associated with less hepatotoxicity and have better adherence and higher treatment completion rates (owing to the reduced duration) [9]. Very recently, an ultra-short-course regimen of daily isoniazid and rifapentine for 28 days (referred to as 1HP) was shown to have a similar efficacy to 9 months of IPT among people with HIV with evidence of latent infection or living in high-burden countries [14]. This condensed regimen, when it becomes available, is likely to have high acceptance among people living with HIV and HIV programs because of the substantially shortened treatment duration and because daily treatment may be more forgiving of poor adherence. 1HP and 3HP may be given safely with efavirenz- and dolutegravir-based antiretroviral therapy, respectively [15,16]. The price of rifapentine is currently a large barrier to scaling up rifapentine-based regimens. The high price of rifapentine is due to the low demand for 3HP and lack of competition from generic manufacturers. Rifapentine is currently manufactured by a sole supplier, the originator (Sanofi). Strategies to reduce the price of rifapentine include increasing the number of people starting 3HP and bringing generic rifapentine single- and fixed-dose combination tablets for adults and children to market. Findings from a recent modelling study suggest that short-course TPT regimens in high-burden countries may be cost effective, depending on the price of rifapentine, treatment completion rates, and willingness to pay [17]. Unitaid funded the IMPAACT4TB project to implement a strategy to reduce the price of rifapentine and catalyse scale-up of 3HP in select high-burden countries. Funding from the President's Emergency Plan for AIDS Relief, The Global Fund to Fight AIDS, Tuberculosis and Malaria, and the United States Agency for International Development, along with technical assistance from WHO, will be required to achieve global scale-up of 3HP.

## Preventing TB to end TB

Modelling studies suggest that by integrating TPT for persons at high risk of developing TB into a comprehensive epidemic control strategy that implements quality services for finding and treating TB disease, strengthening linkages to HIV and child services will accelerate progress towards TB elimination [18]. Validated biomarkers to help identity those at high risk for disease progression would help reduce the number needed to treat and is an area of ongoing investigation.

To end TB once and for all in high-burden countries, we need to prevent cases of TB disease arising from the large reservoir of latently infected persons and thereby interrupt transmission. High-burden countries need to heed the call of the United Nations High Level Meeting on TB to prioritise scaling up TPT. We now have new short- and ultra-short-course regimens that address many of the barriers to scaling up IPT. The time has come to scale up the new short-course TPT regimens in high-burden countries in order to interrupt transmission and end the TB epidemic.

## Abbreviations

IPT: isoniazid preventive therapy
MDR: multidrug-resistant
TB: tuberculosis
TNF: tumour necrosis factor
TPT: TB preventive therapy
WHO: World Health Organization
1HP: 28 days daily isoniazid and rifapentine
3HP: 3 months weekly high-dose isoniazid and rifapentine
